# Next Generation Sequencing for Long Non-coding RNAs Profile for CD4^+^ T Cells in the Mouse Model of Acute Asthma

**DOI:** 10.3389/fgene.2019.00545

**Published:** 2019-06-07

**Authors:** Zhengxia Wang, Ningfei Ji, Zhongqi Chen, Chaojie Wu, Zhixiao Sun, Wenqin Yu, Fan Hu, Mao Huang, Mingshun Zhang

**Affiliations:** ^1^Department of Respiratory and Critical Care Medicine, The First Affiliated Hospital of Nanjing Medical University, Nanjing, China; ^2^Department of Infectious Disease, Taizhou People’s Hospital, Taizhou, China; ^3^State Key Laboratory of Reproductive Medicine, Nanjing Medical University, Nanjing, China; ^4^NHC Key Laboratory of Antibody Technique, Nanjing Medical University, Nanjing, China; ^5^Department of Immunology, Nanjing Medical University, Nanjing, China

**Keywords:** asthma, CD4^+^ T lymphocyte, long non-coding RNA, mRNA, next-generation sequencing

## Abstract

**Background and Aims:**

Although long non-coding RNAs (lncRNAs) have been linked to many diseases including asthma, little is known about lncRNA transcriptomes of CD4^+^ T cells in asthma. The present study aimed to explore the lncRNAs profile in the CD4^+^T cells from the mouse model of acute asthma.

**Methods:**

Next generation sequencing for lncRNAs and mRNAs was performed on CD4+ T cells from asthma and control mice. Gene ontology (GO) and kyoto encyclopedia of genes and genomes (KEGG) pathway analyses were performed to predict the functions and signal pathways for the aberrant lncRNAs. The selected lncRNAs were further measured using quantitative real-time PCR (polymerase chain reaction) and observed in the fluorescence *in situ* hybridization (FISH). The lncRNA–mRNA co-expression network was constructed via Pearson’s correlation coefficient and Cytoscape 3.6.

**Results:**

Next generation sequencing revealed 36 up-regulated lncRNAs and 98 down-regulated lncRNAs in acute asthma compared with controls. KEGG pathway analysis showed that cytokine-cytokine receptor interaction had the highest enrichment scores. A co-expression network was constructed in which 23 lncRNAs and 301 mRNAs altered formed a total of 12424 lncRNA and mRNA pairs. To validate the RNA sequencing results, we measured the 4 different lncRNAs using qPCR. The lncRNA fantom3_9230106C11 was significantly reduced in CD4+ T cells of asthma. Bioinformatics analysis showed that lncRNA fantom3_9230106C11 had the potential to interact with many miRNAs and transcription factors related to Th2 differentiation.

**Conclusion:**

This study provided the first evidence for different expression of lncRNAs of CD4+T cells in asthma and may serve as a template for further, larger functional in-depth analyses regarding asthma molecular lncRNAs.

## Introduction

Allergic asthma is a type of asthma provoked by allergens, including pollen, animal dander, fungal spores, or house dust mites (HDM) (Takyar et al., 2013; Hondowicz et al., 2016). Once processed and presented by dendritic cells, allergens promote the differentiation and expansion of Th2 cells, releasing type 2 cytokines (IL-4, IL-5, IL-13) and expressing the master transcription factor GATA-3. Th2 cells orchestrate eosinophil maturation and survival, airway hyperresponsiveness, and B cell isotype switching to IgE (Holgate, 2012). Consequently, Th2 cells play crucial roles in the cascade reactions of allergic asthma.

Non-coding RNAs emerged as essential players in Th2 cell differentiation and allergic asthma. Recently, we reported the aberrant microRNA (miRNA) profile in CD4^+^ T cells from a murine model of acute asthma (Liu et al., 2018). In addition to miRNAs, long non-coding RNAs (lncRNAs), which comprise 200 nucleotides lacking putative open reading frames, may regulate CD4^+^ T cell differentiation in asthma (Zhang F. et al., 2017). In allergic asthma, the lncRNA profile has been documented in primary airway smooth muscle cells, CD8^+^ T cells or blood samples (Tsitsiou et al., 2012; Austin et al., 2017; Zhu et al., 2018). To our knowledge, lncRNA expression in the CD4^+^ T cells in asthma remains elusive.

In this study, we performed next-generation sequencing and data mining to investigate the whole spectrum of transcriptional signatures (mRNAs and lncRNAs) of CD4^+^ T cells in the murine model of acute asthma. Some lncRNAs were measured in CD4^+^ cells *ex vivo* or in Th2 cells *in vitro* using real-time quantitative reverse transcription PCR (qRT-PCR) and fluorescence *in situ* hybridization (FISH). Moreover, cross-talk between mRNAs and lncRNAs associated with CD4^+^ T cell differentiation was revealed.

## Materials and Methods

### Establishment of the Model of Acute Asthma

Specific pathogen-free female C57BL/6J mice (18 to 22 g) aged 6 to 8 weeks were obtained from the College of Veterinary Medicine Yangzhou University (Yangzhou, China). All experiments that involved animal and tissue samples were performed in accordance with the guidelines and procedures approved by the Institutional Animal Care and Use Committee of Nanjing Medical University (IACUC-1709011).

The model of acute asthma was established as described previously (Liu et al., 2018). Briefly, the asthmatic mice (*n* = 3) were sensitized on days 0 and 14 by the intraperitoneal injection of 20 μg ovalbumin (OVA) (Grade V, Sigma-Aldrich) emulsified in 2 mg aluminum hydroxide gel (InvivoGen, San Diego, CA, United States) in a total volume of 200 μl. These sensitized mice were exposed to aerosolized 1% OVA in sterile saline for 30 min from day 20 to day 22, consecutively. The control subjects (*n* = 3) were sensitized and challenged using the same protocol as used for saline alone. All mice were monitored daily and were alive before sacrifice.

Twenty-four hours after the final challenge, lung function was evaluated by the direct measurement of lung resistance and dynamic compliance in restrained, tracheostomized, mechanically ventilated mice via the FinePointe RC System (Buxco Research Systems, Wilmington, NC, United States) under general anesthesia as described previously (Kerzerho et al., 2013). The sera were collected to measure total IgE using an ELISA kit according to the manufacturer’s instructions (eBioscience, Thermo Fisher Scientific, United States). To determine lung tissue inflammation, the right upper lung lobe was removed, fixed, dehydrated and embedded in paraffin. The fixed embedded tissues were cut into 5 μm sections on a Leica model 2165 rotary microtome (Leica, Nussloch, Germany), and the tissue slides were stained with hematoxylin and eosin (H&E).

### CD4^+^ T Cell Purification From the Spleen

Mice were anesthetized by i.p. injection of a mixture of 10 mg/kg xylazine (MTC Pharmaceuticals, Cambridge, ON, Canada) and 200 mg/kg ketamine hydrochloride (Rogar/STB, London, ON, Canada). The anaesthetized mice were sacrificed with cervical dissociation. The spleen was removed, ground and prepared into single cell suspensions. CD4^+^ T cells in the spleen were sorted using CD4 (L3T4) micro beads (130-049-201, Miltenyi Biotec, United States). Briefly, the single cell suspensions were incubated with CD4 (L3T4) micro beads. The magnetically labeled cells were flushed and collected. Finally, the purity of CD4^+^ T cells was quantified using anti-CD4-APC antibodies (17-0041-81, eBioscience).

### RNA Isolation, Library Preparation, and Sequencing

Total RNA was isolated using the miRNeasy Mini Kit (Qiagen, Germany) and stored at -80°C until use. RNA purity was assessed using the ND-1000 Nanodrop. Each RNA sample had an A260:A280 ratio above 1.8 and A260:A230 ratio above 2.0. RNA integrity was evaluated using the Agilent 2200 TapeStation (Agilent Technologies, United States) and each sample had the RIN above 7.0. Briefly, rRNAs were removed from Total RNA using Epicenter Ribo-Zero rRNA Removal Kit (illumina, United States) and fragmented to approximately 200bp. Subsequently, the purified RNAs were subjected to first strand and second strand cDNA synthesis following by adaptor ligation and enrichment with a low-cycle according to instructions of NEBNext^®^ Ultra^TM^ RNA Library Prep Kit for Illumina (NEB, United States).

The purified library products were evaluated using the Agilent 2200 TapeStation and Qubit^®^ 2.0 (Life Technologies, United States) and then diluted to 10 pM for cluster generation *in situ* on the pair-end flow cell followed by sequencing (2 × 150 bp) on HiSeq 3000. The clean reads were obtained after removal of reads containing adapter, ploy-N and at low quality from raw data. Sequence data were mapped to mouse reference genome mm10 with TopHat v2.0.13. gfold v1.1.2 was subsequently employed to count the number of reads mapped to each gene. Differential expression was assessed by DEGseq using RPKM as input. Differentially expressed genes were chosen according to the criteria of fold change >2 and adjusted *P*-value <0.05. All the differentially expressed genes were used for heat map analysis and KEGG enrichment analyses. For KEGG enrichment analysis, a *P*-value <0.05 was used as the threshold to determine significant enrichment of the gene sets.

### Bioinformatics Data Analysis and Data Mining

The transcriptome was assembled using Cufflinks and Scripture based on the reads mapped to the reference genome. The assembled transcripts were annotated using the Cuff compare program from the Cufflinks package. The unknown transcripts were used to screen for putative lncRNAs. Three computational approaches, including CPC/CNCI/Pfam, were combined to sort non-coding RNA candidates from putative protein-coding RNAs in the unknown transcripts. Putative protein-coding RNAs were filtered out using a minimum length and exon number threshold. Transcripts with lengths greater than 200 nt and with more than two exons were selected as lncRNA candidates and further screened using CPC/CNCI/Pfam, which distinguished protein-coding genes from non-coding genes.

The UCSC genome browser was used to locate lncRNA fantom3_9230106C11. The predicted potential target genes whose loci were within a 10-kb window upstream or downstream of the given aberrantly expressed lncRNA were considered *cis*-regulated genes. Other genes in the co-expression network were identified as *trans-*regulated according to complementary base pairing by LncTar (Li et al., 2015). In addition, Bibiserv (Sczyrba et al., 2003) and RNA22 (Miranda et al., 2006) were used to predict the interactive miRNAs. Different mRNAs in the our NGS were compared with known genes about asthma in the human disease database Malacards associated with asthma^1^ and asthma-associated genes^2^ using Wayne chart^3^.

### CD4^+^ T Cell Differentiation *in vitro*

Naive CD4^+^ T cells in the spleen were sorted using a Naive CD4^+^ T Cell isolation kit (130-104-453, Miltenyi Biotec, United States). Briefly, the single cell suspensions were incubated with biotin-antibody cocktails, which depleted the non-T cells and memory CD4^+^ T cells. The untouched naive CD4^+^ T lymphocytes were seeded onto anti-CD3 (85-16-0031-85, eBioscience, 1 mg/ml) precoated 96-well plates. For Th1 polarization, naive CD4^+^ T cells were further stimulated with anti-CD28 (85-16-0281-85, eBioscience, 2 μg/ml), IL-2 (212-12, PeproTech, 20 ng/ml), IL-12 (210-12, PeproTech, 50 ng/ml) and anti-IL-4 (85-16-7041-85, eBioscience, 10 μg/ml). For Th2 polarization, naive CD4^+^ T cells were further stimulated with anti-CD28 (85-16-0281-85, eBioscience, 2 μg/ml), IL-2 (212-12, PeproTech, 20 ng/ml), IL-4 (214-14, PeproTech, 200 ng/ml), anti-IFN-γ (85-16-7311-85, eBioscience, 10 μg/ml), and anti-IL-12 (85-16-7123-85, eBioscience, 10 μg/ml). Half of the complete RPMI-1640 medium (16000-044, Gibco, United States) with 10% FBS (Gibco, United States) was replaced with fresh medium to maintain the cytokine environments. The CD4^+^ T cells were harvested and analyzed on day 5.

### Flow Cytometry Analysis *ex vivo* and *in vitro*

For nuclear protein assays, spleens from animal experiments were prepared as single-cell suspensions. After RBC (red blood cell) lysis, the cells were stained with CD16/CD32 FcR (Fc Receptor) blocking antibody, Fixable Viability Dye eFluor^TM^ 506 and anti-CD4-APC (eBioscience, 17-0041-83). For intracellular staining, the cells were fixed and permeabilized (eBioscience, 00-5523-00) according to the manufacturer’s instructions, and then intracellular products were stained. Flow cytometry was performed with anti-Gata3-PE-Cy7 (BD Biosciences, 560405), anti-T-bet-PE-Cy7 (eBioscience, 25-5825-80) and isotype controls.

For cytokine assays, T cells were restimulated on day 5 of culture for 5 h with 20 nM PMA (70-CS1001, Multiscience, China) and blocked with BFA (70-CS1002, Multiscience, China). After 5 h, the cells were harvested, gently washed with PBS containing 1% bovine serum albumin (BSA) and fixed in IC fixation buffer (00-822-49, eBioscience, United States). The fixed cells were permeabilized with permeabilization buffer (00-8333-56, eBioscience, United States) and stained with PE-anti-IFN-γ (85-12-7311-84, eBioscience, United States), PE-anti-IL4 (85-12-7041-83, eBioscience, United States) or the isotype control. The stained CD4^+^ T lymphocytes were analyzed on the BD FACSCalibur, and the data were analyzed by FlowJo software (TreeStar).

### Real-Time Quantitative Polymerase Chain Reaction

RNAs from cells or tissues were isolated using a miRNeasy mini kit. The cDNA was synthesized using the Prime Script RT Reagent Kit (TaKaRa, Kyoto, Japan) following the manufacturer’s instructions. The quantitative PCR analysis was performed using the CFX96 system (Bio-Rad laboratories) in conjunction with ready-to-use fast-start SYBR Premix Ex Taq II (TaKaRa, Kyoto, Japan). The cycling conditions were 95°C for 30 s, followed by 95°C for 5 s and 60°C for 30 s for up to 40 cycles and dissociation at 95°C for 15 s, 60°C for 30 s and a final extension at 95°C for 15 s. The relative abundance of gene targets was determined by the comparative CT (cycle threshold) number normalized against tested β-actin comparative CT. The primers used are shown in Table 1, which were designed by RiboBio Institute (Guangzhou, China) or from primerbank by using primer5.

**Table 1:** Primers used in qRT-PCR.

Name	Sequence
β-actin-F	GAGAAGCTGTGCTATGTTGCT
β-actin-R	CTCCAGGGAGGAAGAGGATG
fantom3_9230106C11-F	CTCTGTCCTGGAAAGTGGTGTC
fantom3_9230106C11-R	TGCTGCCAAGCTAGATGGTC
fantom3_4933428M03-F	TGTCATGCTGTGGTAACAGTGA
fantom3_4933428M03-R	ACACCAGGTAGATATGCAAGGAA
fantom3_F630107E09-F	TTCCTGTTGCCCCAACTGTAG
fantom3_F630107E09-R	GGCAGGGAGTCTTCTACTTCC
T-bet-F	AATCGACAACAACCCCTTTG
T-bet-R	AACTGTGTTCCCGAGGTGTC
IFN-γ-F	ACAATGAACGCTACACACTGC
IFN-γ-R	CTTCCACATCTATGCCACTTGAG
GATA3-F	GAACCGCCCCTTATCAAG
GATA3-R	CAGGATGTCCCTGCTCTCCTT
IL-4-F	AACTCCATGCTTGAAGAAGAACTC
IL-4-R	CCAGGAAGTCTTTCAGTGATGTG


### Correlation and Co-expression Analysis

The co-expression analysis was based on Pearson’s correlation coefficient. Considering the influence of random factors, it is found that using Pearson correlation coefficient only is not strict. Therefore, when the number of samples is less than eight, due to the small number of samples, we use the mixed washing method to calculate the *P*-value to further screen.

The co-expression relationship was screened according to Pearson correlation coefficient and *P*-value. Differentially expressed lncRNAs and mRNAs with fold changes ≥2 and *p* < 0.05 were analyzed. For each lncRNA-mRNA pair, the Pearson correlation (COR) was calculated to identify significantly correlated pairs. The Pearson correlation value cutoff was 0.95 and *p* < 0.05. To create a visual representation, a lncRNA-mRNA regulatory network was constructed using Cytoscape 3.6.

### Fluorescence *in situ* Hybridization (FISH)

Fluorescence *in situ* hybridization assays were performed using the Ribo^TM^ Fluorescent *in situ* Hybridization Kit and the Ribo^TM^ lncRNA FISH Probe Mix (Ribo, Guangzhou, China) according to the manufacturer’s protocols. Briefly, the cells were fixed in 4% formaldehyde for 10 min and then washed with PBS. The cells were incubated with 20 μmol/L lncRNA FISH probe mix at 37°C overnight. After washing, the FISH preparations were counterstained with DAPI observed in confocal microscopy for appropriate fluorescence filter sets (Zeiss, Oberkochen, Germany). The lncRNA probe labeled with Cy3 was designed and synthesized by RiboBio Co., Ltd., Ribo^TM^ U6 and Ribo^TM^ 18S were used as reference controls for the subcellular localization of lncRNA.

### Statistical Analysis

The statistical analysis was performed using GraphPad Prism version 5.0 (GraphPad, San Diego, CA, United States). The results for variables that were normally distributed are displayed as the means ± SEM. An ANOVA was performed to establish equal variance, and a 2-tailed Student’s *t*-test with Bonferroni correction was applied to determine statistical significance, which was defined as *P* < 0.05.

## Results

### Establishment of an Acute Model of Asthma

**FIGURE 1:**
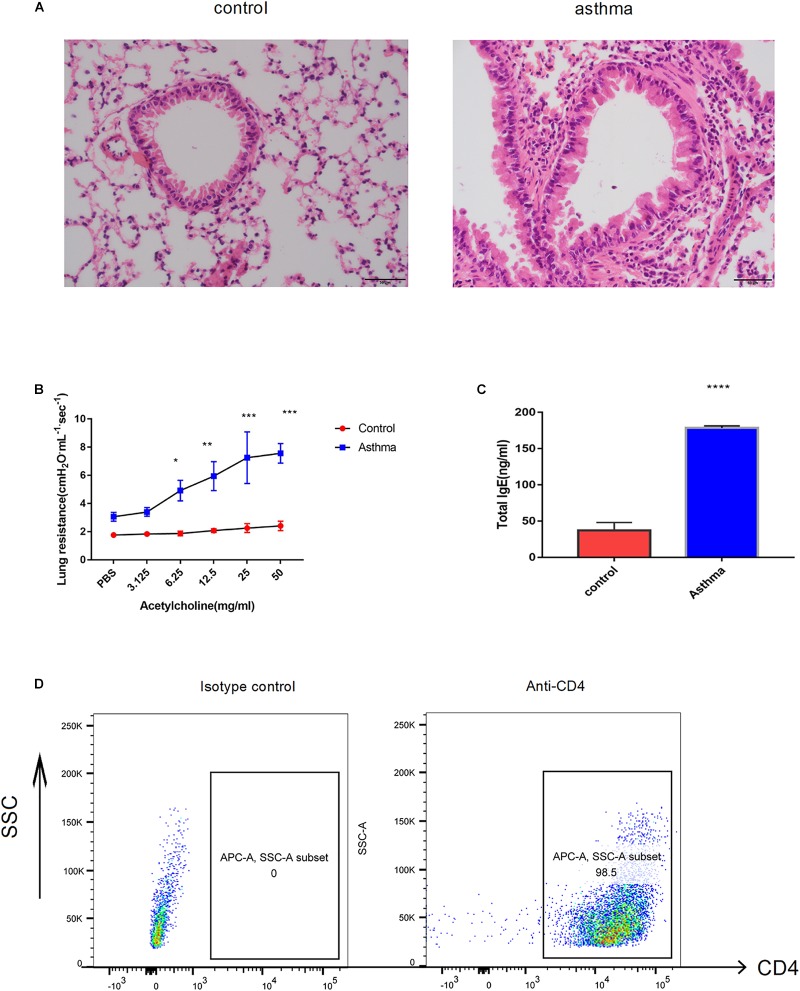
Ovalbumin (OVA)-induced acute model of asthma. **(A)** Pulmonary inflammatory response was increased in the mice challenged with OVA, bar = 20 μm. **(B)** The airway hyperresponse was elevated in the mice from the asthma group, ^∗^*p* < 0.05 **(C)**. Sera IgE was significantly increased in the mice from the asthma group, ^∗^*p* < 0.05. **(D)** CD4^+^ T cells magnificently sorted from the spleen were validated by flow cytometry. Representative results of two independent experiments with five mice in each group, ^∗∗^*p* < 0.01, ^∗∗∗^*p* < 0.01, ^∗∗∗∗^*p* < 0.0001.

As the well-established murine model of asthma, OVA challenge provoked evident pulmonary inflammation, airway hypersensitivity and higher IgE in serum (Figure 1A–C). CD4^+^ T cells were magnetically sorted from the spleen, and the purity was validated by flow cytometry. As shown in Figure 1D, 98.5% of the cells were CD4 positive (Figure 1D).

### LncRNA Profile of CD4^+^ T Cells in Asthma

**FIGURE 2:**
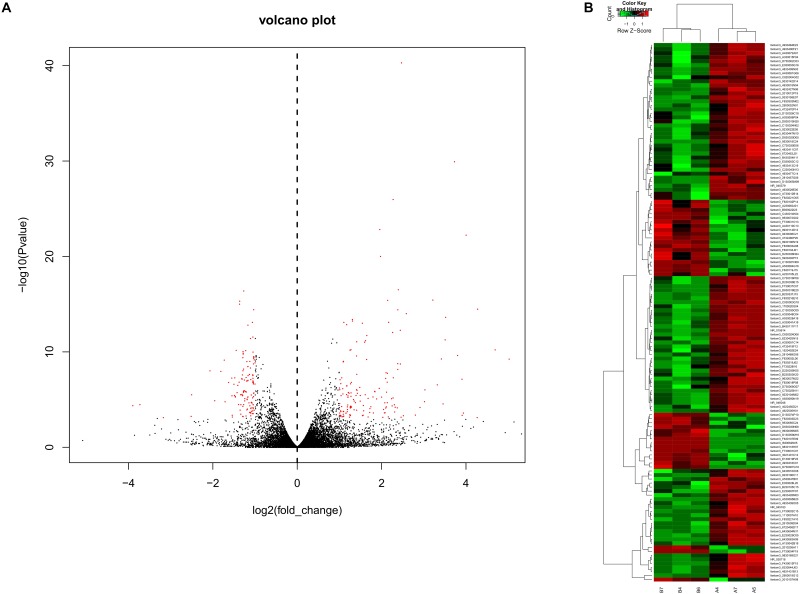
Volcano plots and heat maps of lncRNA expression between the asthma and control groups. **(A)** Volcano plot assessment of lncRNA expression in CD4^+^ T cells between the asthma and control groups. Red dots represent different lncRNAs (*p* < 0.05) showing fold changes >=2 or <= -2. **(B)** Heat map analysis of differentially expressed lncRNAs between the asthma and control groups. Green indicates low expression, and red indicates high expression. A4, A5, and A7 were the control group; B4, B6, and B7 were CD4^+^ T cells from the asthma group.

To obtain a global overview of CD4^+^ T cells in the asthma transcriptome, we constructed and sequenced 6 RNA-Seq libraries, including controls (*n* = 3) and asthma mice (*n* = 3). All of the data have been submitted to Sequence Read Archive (SRA ^4^) with the accession number of PRJNA540404. In total, 134 lncRNAs were significantly altered in expression, including 98 downregulated lncRNAs and 36 upregulated lncRNAs (Figure 2). The top 10 decreased lncRNAs and top 10 increased were listed in Table 2.

**Table 2:** Top 20 lncRNAs in CD4^+^ T cells between asthma and control groups.

LncRNA	control	asthma	*P*-value
fantom3_E230007F07	8.78	0.61	0.0006
fantom3_4933428M03	14.37	1.08	< 0.0001
fantom3_B230105C15	10.41	0.99	0.0005
fantom3_E030028L20	12.64	1.76	0.0003
fantom3_A530047B01	21.82	4.05	< 0.0001
fantom3_9230106C11	24.67	5.02	0.0002
fantom3_C130024N02	121.92	26.59	< 0.0001
fantom3_9230022E05	100.13	22.58	0.0001
fantom3_D130005M09	37.46	9.67	< 0.0001
fantom3_6430516O08	25.14	7.87	0.0006
fantom3_F630107E09	3.96	20.46	0.0001
fantom3_9930009M05	3.87	20.74	0.0006
fantom3_9530065C24	4.89	27.20	0.0001
fantom3_D130059M16	3.25	21.04	< 0.0001
fantom3_3021401C12	2.21	23.19	< 0.0001
fantom3_I830062N05	5.59	62.54	< 0.0001
fantom3_F730001C01	4.58	52.60	< 0.0001
fantom3_9830118H07	4.92	58.38	< 0.0001
fantom3_E130019F23	1.31	16.65	< 0.0001
fantom3_4930418C01	0.33	9.44	< 0.0001


*The P-value indicates the difference between the control and asthma groups.*

### The mRNA Profile of CD4^+^ T Cells in Asthma

**FIGURE 3:**
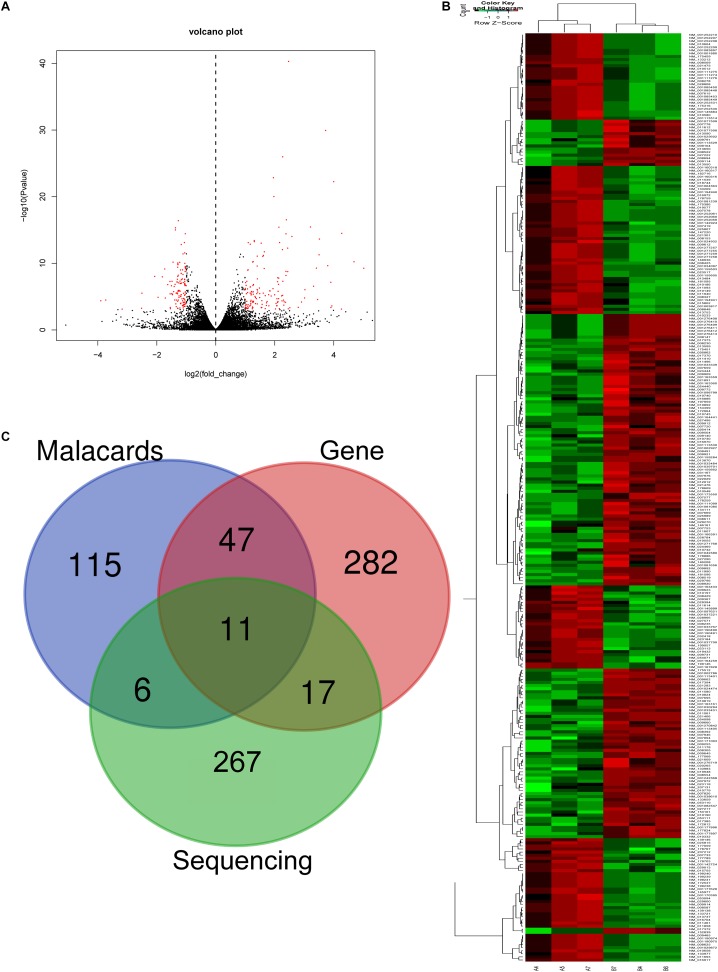
Volcano plots and heat map of mRNA expression between asthma and control groups. **(A)** Volcano plot assessment of gene expression in CD4^+^ T cells between asthma and control groups. Red dots represent different mRNAs (*p* < 0.05) showing fold changes >=2 or <= -2. **(B)** Heat map analysis of differentially expressed mRNAs between the asthma and control groups. Green indicates low expression, and red indicates high expression. A4, A5, and A7 were the control groups; B4, B6, and B7 were CD4^+^ T cells from the asthma group. **(C)** The Venn diagram of genes about asthma from sequencing compared with gene database and malacards database.

In RNA sequencing, we explored not only the lncRNAs but also the mRNAs from the same samples. With respect to mRNAs, 141 mRNAs were significantly downregulated and 160 mRNAs were significantly upregulated in the CD4^+^ T cells from asthma (Figure 3). The top 10 decreased and top 10 increased mRNAs are listed in Table 3, including reduced Kbtbd12, Hunk, and Slc6a1 and elevated Ear7, Ear6, and Epx. Among of the 301 differently expressed mRNAs, 17 mRNAs were in the human disease database Malacards associated with asthma (see text footnote 1), 28 mRNAs were in the asthma-associated genes^5^. More importantly, 11 mRNAs were overlapped in our sequencing data, MalaCards database and asthma-associated genes, including IL4, IL-10, MMP9, VCAM-1, Il1rl1, Alox5, Il1rn, Ccr3, Cysltr1, Epx, and Ccl24.

**Table 3:** Top 20 mRNAs in CD4^+^ T cells between asthma and control groups.

mRNAs	control	asthma	*P*-value
Kbtbd12	11.39	0.76	< 0.0001
Kbtbd12	14.37	1.08	< 0.0001
Hunk	13.10	1.45	0.0008
Slc6a1	24.04	4.20	0.0006
Vsig4	25.70	4.53	< 0.0001
Fstl4	57.58	13.73	< 0.0001
Col19a1	32.03	8.25	< 0.0001
Trim30d	121.32	34.58	< 0.0001
Trim30d	94.45	28.47	< 0.0001
Agmo	44.13	13.48	< 0.0001
Prg2	22.40	296.72	< 0.0001
F10	2.23	31.00	< 0.0001
Ascl2	2.65	39.68	< 0.0001
9830107B12Rik	0.97	14.54	0.0004
9830107B12Rik	0.97	14.86	0.0003
Irg1	5.78	92.58	< 0.0001
9830107B12Rik	0.67	12.84	0.0008
Epx	4.59	88.82	< 0.0001
Ear6	1.02	26.46	< 0.0001
Ear7	0.69	22.45	< 0.0001


*The P-value indicates the difference between the control and asthma groups.*

### The lncRNA-mRNA Co-expression Network

**FIGURE 4:**
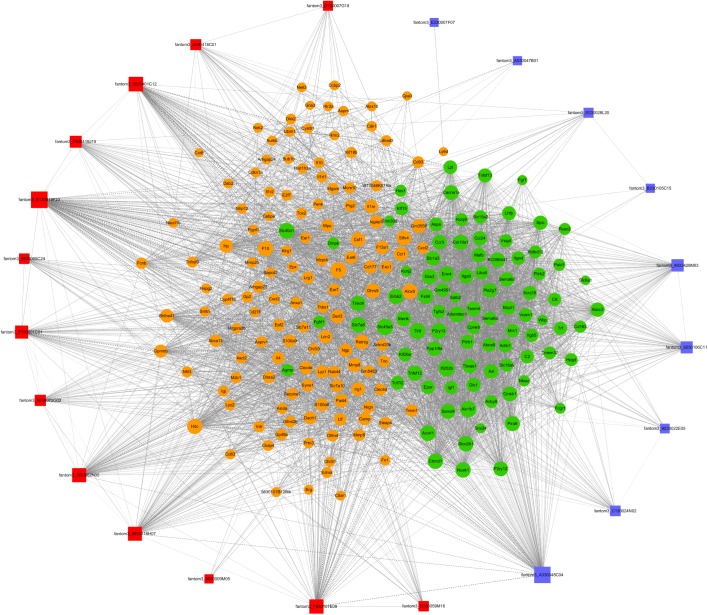
LncRNA–mRNA co-expression networks. Twenty-three lncRNAs and 301 mRNAs were included in the co-expression network | COR| >0.95; *p* < 0.05). The red boxes indicate upregulated lncRNAs, and the blue boxes indicate downregulated lncRNAs. The green nodes indicate downregulated mRNAs, and the yellow nodes indicate upregulated mRNA. The value of “degree” indicates the size of the boxes or nodes. The width of the lines denotes the Pearson correlation coefficient, where the full line indicates a positive correlation and the imaginary line indicates a negative correlation.

To further examine the function of these differentially expressed lncRNAs in CD4^+^ T cells in asthma, we constructed an lncRNA-mRNA co-expression network between 301 differentially expressed mRNAs and 23 differentially expressed lncRNAs. The results showed that the co-expression network comprised 12424 connections between lncRNAs and mRNAs. Notably, fantom3_4933428M03 and fantom3_9230106C11 exhibited a high degree of connectivity, suggesting that these two lncRNAs may play key roles (Figure 4 and Table 4). Moreover, our results highlighted the potential internal adjustment correlations between the differentially expressed lncRNAs and mRNAs in CD4^+^ T cells between the asthma and control groups.

**Table 4:** The “degree” of lncRNAs in the co-expression network.

lncRNA	style	Degree
fantom3_3021401C12	Up	126
fantom3_4930418C01	Up	43
fantom3_9230022E05	Down	102
fantom3_9230106C11	Down	86
fantom3_9530065C24	Up	30
fantom3_9530072G02	Up	39
fantom3_9830118H07	Up	99
fantom3_A330048C04	Down	160
fantom3_C130024N02	Down	39
fantom3_D130059M16	Up	33
fantom3_E130019F23	Up	158
fantom3_F630107E09	Up	108
fantom3_F630119J15	Up	52
fantom3_F730001C01	Up	78
fantom3_A530047B01	Down	120
fantom3_I830062N05	Up	94
fantom3_4933428M03	Down	185


*^∗^<30° was not shown.*

### Validation of RNA-Seq Data With Real-Time PCR

**FIGURE 5:**
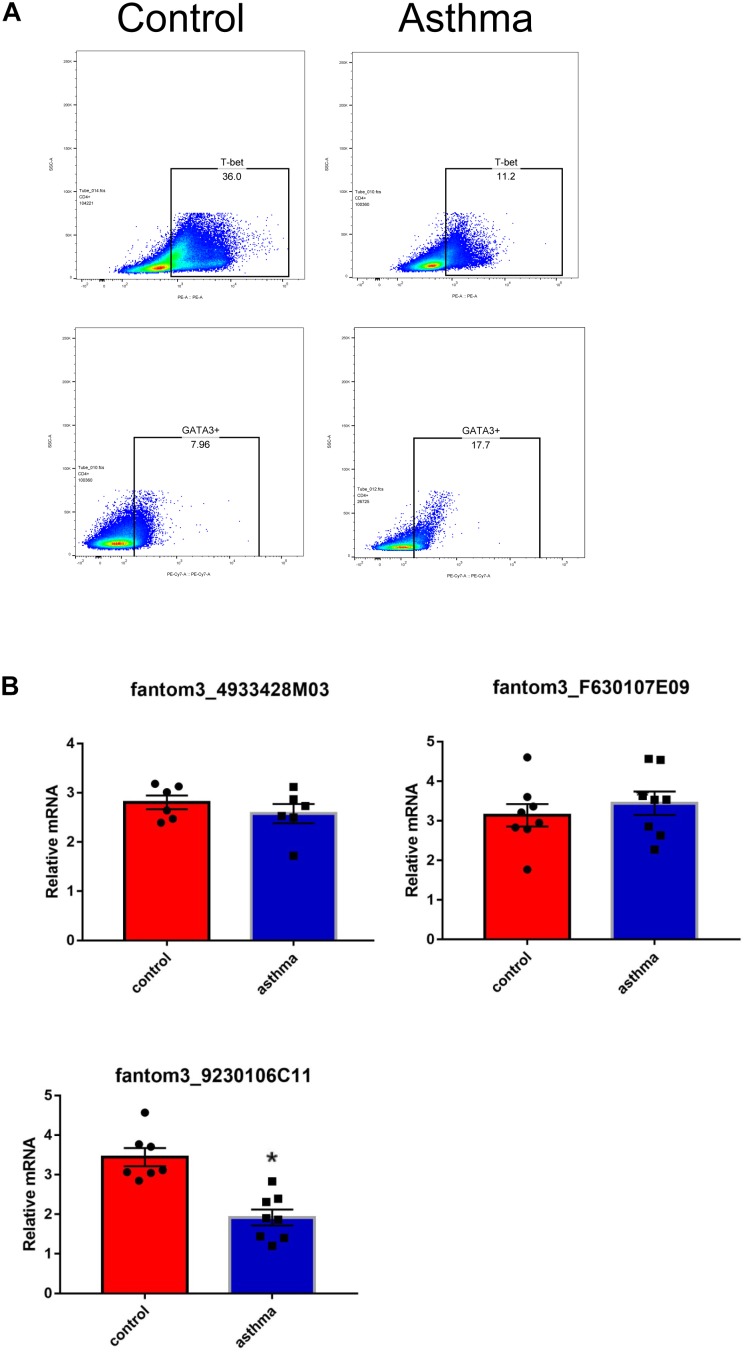
Measurement of selected lncRNAs using qRT-PCR *ex vivo*. **(A)** CD4^+^ T cells in the asthma had lower T-bet and increased Gata-3 expression. Representative result for 3 independent experiments. **(B)** The expression of randomly selected lncRNAs in CD4^+^ T cells between the asthma and control groups were quantified *ex vivo*. ^∗^*p* < 0.05, 6∼8 samples were analyzed in each group.

**FIGURE 6:**
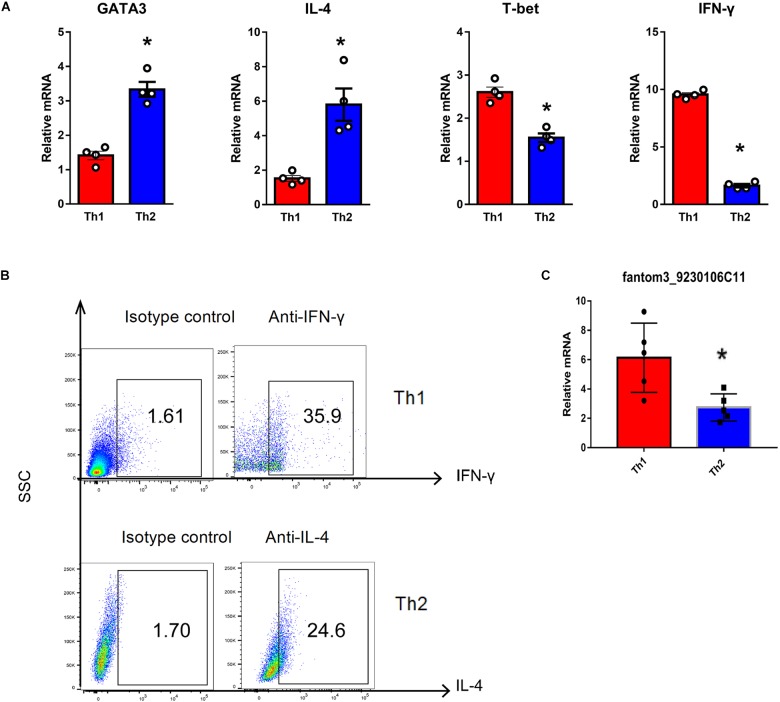
Quantification of LncRNA in Th1 and Th2 cells *in vitro*. **(A)** Typical cytokines and transcription factors for Th1 or Th2 were measured using qRT-PCR. Th1 cells exhibited elevated T-bet and IFN-γ levels. In contrast, Th2 cells expressed higher levels of Gata-3 and IL-4. In each group, 4 samples were analyzed. **(B)** Intracellular cytokines in Th1 and Th2 cells were measured by flow cytometry. Representative result for 3 independent experiments. **(C)** LncRNA fantom3_9230106C11 was measured in Th1 and Th2 cells *in vitro* using qRT-PCR. In each group, 5 samples were analyzed. ^∗^*p* < 0.05.

**FIGURE 7:**
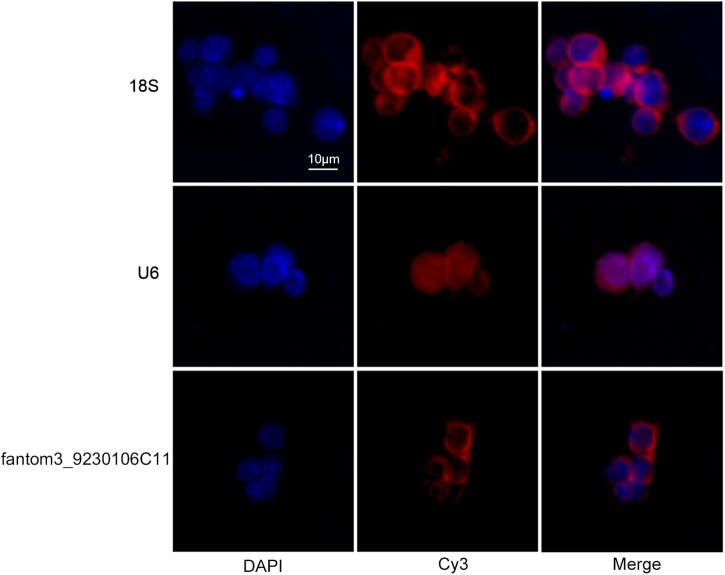
Intracellular location of fantom3_9230106C11 expression in CD4^+^ T cells by FISH. The nucleus was stained with DAPI, and lncRNA was stained with probe-cyt3. Ribo^TM^ U6 and Ribo^TM^ 18S were used as reference controls for subcellular localization.

To confirm the differentially expressed gene data, we further analyzed some dysregulated lncRNAs using qRT-PCR *ex vivo* and *in vitro*. In OVA-induced asthma, Th2 cells orchestrate the pathological cascades reactions. As shown in Figure 5A, CD4^+^ T cells from the asthma group showed higher expression of the Th2 master transcription factor Gata-3 and decreased expression of the Th1 master transcription factor T-bet. Different from the sequencing data, the expression of LncRNA fantom3_4933428M03 or fantom3_F630107E09 was unexpectedly similar in the CD4^+^ T cells from either the asthma or the control group. However, lncRNA fantom3_9230106C11 was in accordance with sequencing data, which was significantly decreased in the asthma CD4^+^ T cells (Figure 5B). To further explore the data reliability, we induced Th1 or Th2 cells *in vitro* (Figure 6A,B). As expected, the expression of lncRNA fantom3_9230106C11 was significantly decreased in Th2 cells (Figure 6C). FISH assays indicated that lncRNA fantom3_9230106C11 was localized in the cytoplasm of CD4^+^ T cells (Figure 7).

### Prediction of lncRNA fantom3_9230106C11 Targets

The above results indicated that lncRNA fantom3_9230106C11 may be involved in Th2 cell differentiation in asthma. LncRNA fantom3_9230106C11 was located at chr6:34412743–34415062 (GenBank: AK033773.1), overlapping with intron 3, exon 4, and intron 4 of Akr1b7 (chr6: 34412362–34423137). Sequence blast analysis shows that lncRNA fantom3_9230106C11 is 99.8% similar with Mus musculus lncRNA URS00009B6C6F^6^. In the NONCODE (current version v5.0) http://www.noncode.org/, Mus musculus lncRNA URS00009B6C6F is renamed with NONMMUT056297.2, which is highly expressed in the hippocampus (Data Source:ERP000591).

**FIGURE 8:**
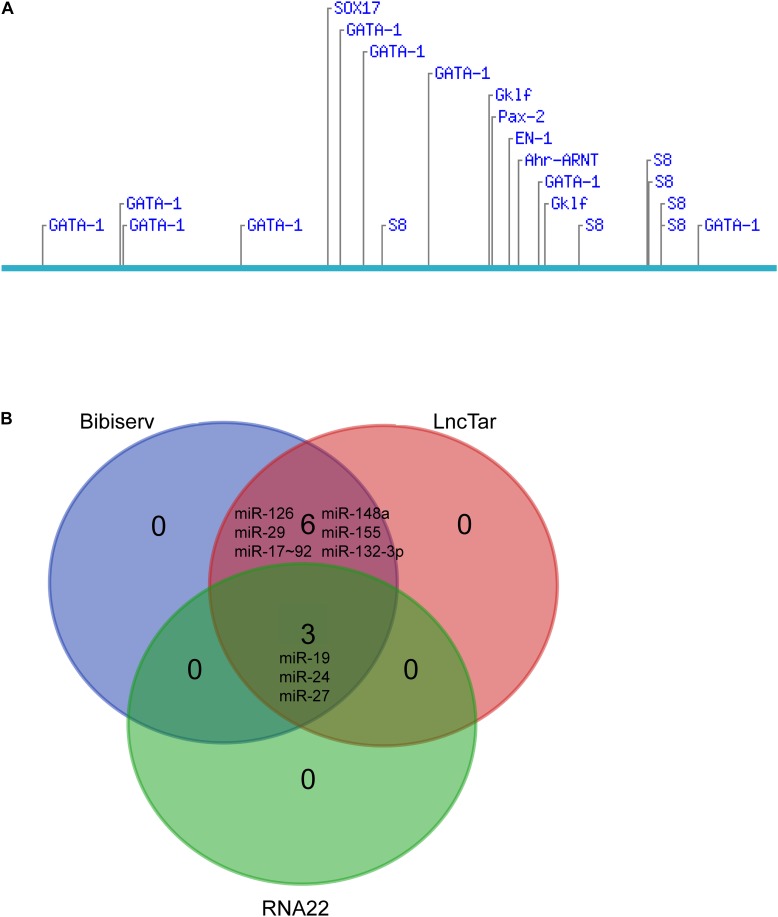
Bioinformatics analysis of lncRNA fantom3_9230106C11. **(A)** Putative transcription factor binding sites found along the lncRNA fantom3_9230106C11 using Consite and TF scores >95%. **(B)** The Venn diagram of potential miRNA candidates using LncTar, Bibiserv, and RNA22.

We further explored the potential candidates that may interact with the lncRNA. Diverse transcription factors, such as T-bet, Gata-3, c-maf, stat4, stat5, stat6, jun-b, Dec-2, IRF4, Notch, Gfi-1, and YY1, may be involved in Th2 differentiation (Hwang et al., 2013; Lee, 2014). We used LncTar (Li et al., 2015) and found that lncRNA fantom3_9230106C11 had no effects on the mRNAs of these transcription factors. The Consite Predication Database (Mahmood et al., 2018) showed that transcription factors Gata1, Gklf, SOX17, S8, and Ahr-ARNT may be bound to the promoter of LncRNA fantom3_9230106C11. In addition to transcription factors, a wide range of miRNAs contribute to Th2 differentiation, i.e., miR-17∼92, miR-29, miR-126, miR-132-3p, miR-148a, mir-24, mir-27, mir-19, and mir-155 were confirmed to regulate Th2 differentiation (Istomine et al., 2016; Pua et al., 2016). In the LncTar, Bibiserv, and RNA 22 analysis, lncRNA fantom3_9230106C11 was predicted to bind with miR-19 and other miRNAs. Collectively, the potential candidates of LncRNA fantom3_9230106C11 may include Akr1b7, Gata1 and miRNAs (Figure 8).

## Discussion

Allergic bronchial asthma is a common chronic inflammation with well-defined pathological features, including intermittent airway hyperresponsiveness, pulmonary eosinophil infiltration, and excessive mucus secretion (Chang et al., 2015). Numerous studies have demonstrated that allergic asthma is driven predominantly by a Th2 type of immune response in both human and mouse models of asthma (Kaiko and Foster, 2011). LncRNAs have been linked with airway smooth muscle cells in asthma (Zhang et al., 2016; Austin et al., 2017; Yu et al., 2017; Zhang X.-Y. et al., 2017). However, to our knowledge, there is no report on lncRNA expression in CD4^+^ T cells in asthma. Therefore, we constructed a mouse model of acute asthma and screened RNA transcripts (mRNAs, lncRNAs) from CD4^+^ T cells via next-generation sequencing.

In our study, we identified 134 lncRNAs and 301 mRNAs abnormally expressed in CD4^+^ T cells of asthma compared with controls. In the mRNA expression pattern analysis, elevated Ear7, Ear6 and Epx were closely associated with eosinophils and type 2 inflammations (Ochkur et al., 2017). IL-4, IL-13, and IL-21 (Lajoie et al., 2014), which promoted Th2 differentiation and allergic asthma, were also significantly increased in the CD4^+^ T cells from the asthma mouse model. However, the Th1 master transcription factor T-bet and the Th2 master transcription factor Gata3 were comparable in CD4^+^ T cells from both control and asthma mice, suggesting that Th2 differentiation in asthma may be independent of Gata3 (O’Shea and Paul, 2010). In the lncRNA profile analysis, the roles of a wide range of lncRNAs with varied expression in asthma CD4^+^ T cells were largely unknown. In the lncRNA and mRNA co-expression network, lncRNAs may regulate diverse mRNAs, including Ear6 and Epx, which are required for Th2 differentiation and asthma etiology.

Using real-time qRT-PCR, we demonstrated that lncRNA fantom3_9230106C11 was decreased in CD4^+^ T cells from asthma mice *ex vivo* and in Th2 cells *in vitro*. By filtering the aberrantly expressed genes located near the lncRNA fantom3_9230106C11s, we found that lncRNAs might regulate the transcription of Akr1b7 in *cis*. lncRNA fantom3_9230106C11s covered intron 3, exon 4, and intron 4 of Akr1b7. *Akr1b7* encodes aldose reductase-related protein 1, which may catalyze xenobiotic aromatic aldehydes (Liu et al., 2009). The roles of *Akr1b7* in Th2 differentiation or asthma, however, remain uncertain. The bioinformation prediction indicated that the transcription factors Gata1, Gklf, and Ahr-ARNT may be potential candidates for lncRNA fantom3_9230106C11. Gata1 served as a surrogate for Gata3 in its canonic role of programming Th2 gene expression (Sundrud et al., 2005). Gklf (gut-enriched Krüppel-like factor; or Kruppel-like factor 4, KLF4) was required in Th2 cell responses *in vivo* (Tussiwand et al., 2015). Ahr-ARNT, which may regulate IL-33, IL-25, and TSLP, was closely associated with allergic severe asthma (Weng et al., 2018). Moreover, considering that lncRNA fantom3_9230106C11 resides in the cytoplasm, it may interact with miR-19 and other miRNAs, which are closely associated with Th2 differentiation.

Our study was not without limitations. First, BALB/c and C57BL/6 mouse strains are the two most commonly used in asthma models. Compared with the C57BL/6 mice used in this study, BALB/c mice were more prone to a Th2 immune response (Gueders et al., 2009). Although allergic asthma was Th2-dominated, Th1 and other CD4^+^ T subpopulations were also involved in the initiation and aggravation of disease. Therefore, the C57BL/6 mouse model was well recognized in the recapitulation of disease features in asthma. Second, spleen CD4^+^ T cells rather than lung CD4^+^ T cells were analyzed in the present study. We tried to sort CD4^+^ T cells from lung parenchyma and pulmonary lymph nodes. However, there were not enough purified CD4^+^ T cells (5^∗^10^6^ from each mouse) to complete the NGS experiment. Previously, adoptive transfer experiments demonstrated that peripheral CD4^+^ T lymphocytes regulate asthma pathogenesis (Cohn et al., 2004; Hubeau et al., 2006). Therefore, we postulated that spleen CD4^+^ T cells may be surrogates for peripheral CD4^+^ T lymphocytes. Third, we have not validated our observations in the clinical samples. LncRNAs are considered poorly conserved across different species (Ma et al., 2013). However, the conservation may be multidimensional (Diederichs, 2014). The expression of aberrant lncRNAs (lncRNA fantom3_9230106C11) in the clinical samples should be evaluated.

## Conclusion

In conclusion, we conducted a comprehensive analysis of lncRNA profiles in CD4^+^ T cells from an asthma model using next-generation sequencing. The co-expression network of lncRNAs and mRNAs was constructed. The present study provided a platform for elucidating the roles of lncRNAs in Th2 differentiation and asthma pathogenesis.

## Ethics Statement

All experiments that involved animal and tissue samples were performed in accordance with the guidelines and procedures approved by the Institutional Animal Care and Use Committee of Nanjing Medical University (IACUC-1709011).

## Author Contributions

MH and MZ conceived the idea and designed the research. ZW, NJ, ZC, and CW conducted the animal and *in vitro* experiments. ZS, WY, and MZ analyzed the results. FH performed the flow cytometry. ZW and MZ wrote the manuscript. All the authors reviewed and approved of the manuscript.

## Conflict of Interest Statement

The authors declare that the research was conducted in the absence of any commercial or financial relationships that could be construed as a potential conflict of interest.

## Footnotes

1 https://www.malacards.org/

2 https://www.ncbi.nlm.nih.gov/gene/?term=asthma

3 http://bioinformatics.psb.ugent.be/webtools/Venn/

4 www.ncbi.nlm.nih.gov/sra">http://www.ncbi.nlm.nih.gov/sra">www.ncbi.nlm.nih.gov/sra

5 https://www.ncbi.nlm.nih.gov/gene

6 https://rnacentral.org/rna/URS00009B6C6F
